# Gut microbiota differs between treatment outcomes early after fecal microbiota transplantation against recurrent *Clostridioides difficile* infection

**DOI:** 10.1080/19490976.2022.2084306

**Published:** 2022-06-05

**Authors:** Shaodong Wei, Martin Iain Bahl, Simon Mark Dahl Baunwall, Jens Frederik Dahlerup, Christian Lodberg Hvas, Tine Rask Licht

**Affiliations:** a National Food Institute, Technical University of Denmark, Kgs Lyngby, Denmark; b Department of Hepatology and Gastroenterology, Aarhus University Hospital, Aarhus N, Denmark

**Keywords:** *Clostridioides difficile*, fecal microbiota transplantation, fidaxomicin, gut microbiota, vancomycin

## Abstract

In fecal microbiota transplantation (FMT) against recurrent *Clostridioides difficile* infection (CDI), clinical outcomes are usually determined after 8 weeks. We hypothesized that the intestinal microbiota changes earlier than this timepoint, and analyzed fecal samples obtained 1 week after treatment from 64 patients diagnosed with recurrent CDI and included in a randomized clinical trial, where the infection was treated with either vancomycin-preceded FMT (*N* = 24), vancomycin (*N* = 16) or fidaxomicin (*N* = 24). In comparison with non-responders, patients with sustained resolution after FMT had increased microbial alpha diversity, enrichment of Ruminococcaceae and Lachnospiraceae, depletion of Enterobacteriaceae, more pronounced donor microbiota engraftment, and resolution of gut microbiota dysbiosis. We found that a constructed index, based on markers for the identified genera *Escherichia* and *Blautia*, successfully predicted clinical outcomes at Week 8, which exemplifies a way to utilize clinically feasible methods to predict treatment failure. Microbiota changes were restricted to patients who received FMT rather than antibiotic monotherapy, indicating that FMT confers treatment response in a different way than antibiotics. We suggest that early identification of microbial community structures after FMT is of clinical value to predict response to the treatment.

## Introduction

*Clostridioides difficile* (formerly *Clostridium difficile*) is a gram-positive, spore-forming, anaerobic bacterium that accounts for a significant proportion of hospital and community-acquired infections.^1^ The symptoms of *C. difficile* infection (CDI) range from diarrhea to pseudomembranous colitis and death. Current guidelines recommend fidaxomicin or vancomycin as first-line treatment for CDI,^2,3^ although antibiotic treatment is often accompanied by a high recurrence rate.^4^

In recent years, fecal microbiota transplantation (FMT) has been implemented as a highly effective treatment of CDI.^5–7^ FMT involves the transfer and possible engraftment of gut microbes from healthy donors into the intestinal tract of the recipients. This treatment enables the reestablishment of a healthy gut microbial community, inhibits the growth of *C. difficile*, and prevents recurrence of infections.^8^ A successful FMT likely depends on the engraftment of donor microbes and presence of keystone species in the donors,^9^ although the degree of engraftment does not appear to be the primary driver of a successful outcome.^10,11^

Administration of fecal microbiota, *e.g., via* colonoscopy, frequently results in prompt engraftment of donor microbes,^12^ and the clinical outcome of FMT is usually determined 8 weeks after treatment.^13,14^ Although earlier identification of FMT failure would be of great clinical value, only a few studies, not including comparator groups or randomized design, have currently addressed it,^15,16^ and the predictive value of microbiota changes thus remains to be clearly established.

In this study, we aimed to investigate the impact of FMT on the gut microbiota, especially to compare the microbial differences between clinical outcomes and to identify putative microbial differences that potentially could be used to predict successful or nonsuccessful clinical responses to FMT. Additionally, we compared the effects of FMT on the gut microbiota to those inflicted by treatment with vancomycin or fidaxomicin.

## Results

### Taxonomic composition changes post-FMT

For the 24 patients randomized to FMT preceded by vancomycin (FMTv) treatment (**Figure S1**), diarrhea resolution together with a negative *C. difficile* PCR test at Week 8 (W8) occurred in 17/24 (71%).^17^ The most pronounced difference between samples from patients prior to FMT (Week 0, W0) and samples from healthy donors, was a higher relative abundance of Proteobacteria (**Figure S2**). In patients with recurrent CDI, Firmicutes was the most abundant phylum (relative abundance 69.6%), followed by Proteobacteria (23.1%), Bacteroidetes (4.2%), Actinobacteria (1.2%); thus, these four phyla together constituted 98.1% of the community. In donor samples, the four most abundant phyla were Firmicutes (78.4%), Bacteroidetes (19.1%), Actinobacteria (1.3%), and Proteobacteria (0.9%), which had a combined relative abundance of 99.8%.

**Figure 1.**
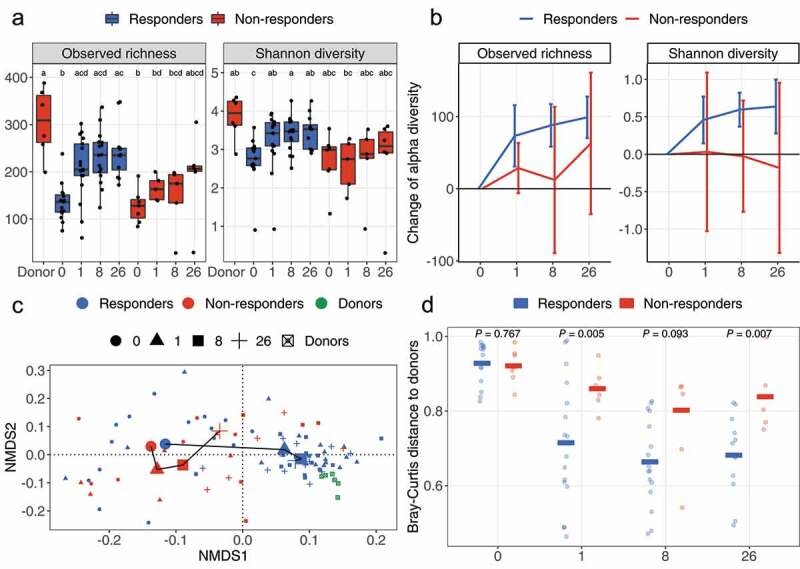
Change of alpha and beta diversity between outcomes. (a) The observed richness and Shannon diversity for responders, non-responders, and donor samples. Alphabet letters are the Tukey’s *post -hoc* test; sharing same letters indicate no significant difference at a level of 0.05. (b) The change of observed richness and Shannon diversity within patients and averaged by outcomes. The error bars refer to the 95% confidence interval of the mean estimated with paired Welch’s t-test. Error bars not overlapping the zero value indicate significant changes compared to pre-FMT (W0). (c) The beta diversity of samples from response and non-response patients assessed with Bray-Curtis distance and visualized with nonmetric multidimensional scaling (NMDS) ordination. Shapes refer to time points or donor. Colors refer to outcomes or donor. Samples are shown with small shapes referring to different time points. The big shapes represent the centroid of sample groups in each condition and connected with lines in the order of time point. (d) Bray-Curtis distance between patient samples and corresponding donor samples. Each dot refers to one distance between a given patient and the processed feces coming from the corresponding donor. The crossbars represent mean value distances. *P* values are obtained with the Welch’s t-test. For (a) and (c), the taxa abundances in fecal samples coming from the same donor (in total six donors provided 24 samples) were averaged so that each donor is represented by a single community.

We then stratified FMTv samples according to treatment response at W8 and assessed the observed richness and Shannon diversity for the responders (*n* = 17) and non-responders (*n* = 7) at W0, Week 1 (W1), W8, and Week 26 (W26) (Figure 1(a)). The highest richness was found in donor samples (309 ± 99, median ± interquartile range [IQR]), while patients at W0 were lowest in richness regardless of future outcomes (136 ± 37 [responders], 128 ± 39 [non-responders]). In responders, the richness increased significantly from W0 to W1 (205 ± 67, adjusted *P-value* [*P_adj_*] = 0.009) and remained stable until W26 (236 ± 64 [W8], *P_adj_* = 0.001; 235 ± 46 [W26], *P_adj_* = 0.001). Conversely, in patients who experienced CDI recurrence following FMT before Week 8, the richness was not significantly increased neither at W1 (163 ± 40, *P_adj_* = 0.126), W8 (175 ± 60, *P_adj_* = 0.476) nor W26 (207 ± 11, *P_adj_* = 0.101), as compared to W0. Shannon diversity followed a similar trend (Figure 1(a)). We also monitored the change of alpha diversity within each patient relative to W0 (Figure 1(b)). Based on a 95% confidence interval of the mean, the microbial richness as well as the Shannon diversity were significantly increased in responders at W1 (73.3 ± 19.8 [richness, mean ± standard error (SE)], *P* = .002; 0.46 ± 0.15 [Shannon], *P* = .007; increase of alpha diversity), W8 (87.8 ± 13.8 [richness], *P* < .001, 0.59 ± 0.11 [Shannon], *P* < .001), and W26 (98.9 ± 13.1 [richness], *P* < .001, 0.64 ± 0.16 [Shannon], *P* = .003), as compared to W0. However, non-responders did not show any significant increase in alpha diversity compared to W0.

Comparison of fecal microbiota based on Bray-Curtis distances (Figure 1(c)) revealed that significant changes occurred in the gut microbiota of responders at W1 compared to W0 (*P_adj_* = 0.002, W1 *vs*. W0), and thereafter the composition remained unchanged (**Table S1**). The microbiota of non-responders did not change at neither W1 (*P_adj_* = 0.612), W8 (*P_adj_* = 0.077), nor W26 (*P_adj_* = 0.117), as compared to W0. This divergence between outcomes at W1 was also reflected by the distance between patients and their corresponding donors (Figure 1(d)) where all patients showed similar distances to donors at W0 (0.93 [responders, mean] *vs*. 0.92 [non-responders, mean], *P* = .767), but thereafter frequently differed between outcomes at W1 (0.71 [responders] *vs*. 0.86 [non-responders], *P* = .005), W8 (0.66 [responders] *vs*. 0.80 [non-responders], *P* = .093) and W26 (0.68 [responders] *vs*. 0.84 [non-responders], *P* = .007).

### Taxonomic composition differences between outcomes

**Figure 2.**
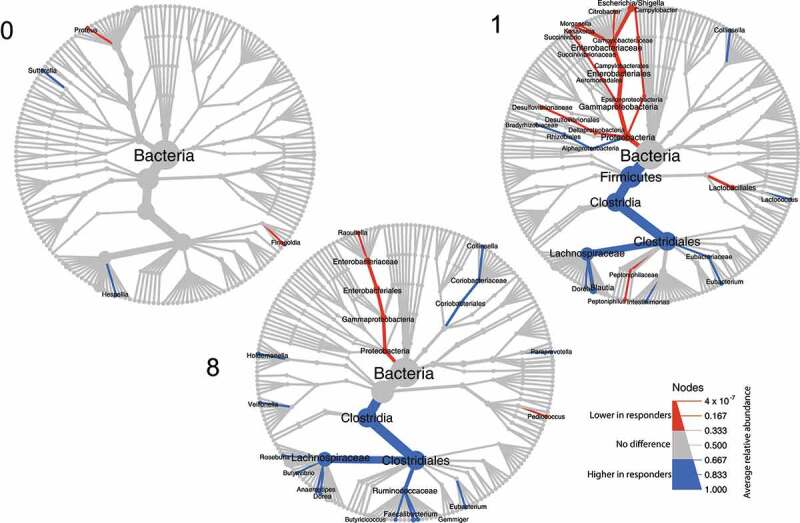
Tree plot showing taxa with different relative abundances between outcomes. The largest node in the center is the kingdom bacteria. Along the tree branch outward, the next node is the phylum level, then followed by class, order, family, and genus. The size of the node is proportional to the mean relative abundance in the corresponding phylogenetic level. A node is labeled when it has significantly different relative abundances (Wilcoxon rank-sum test) between outcomes. Blue refers to higher abundances in responders, red refers to lower abundances in responders. The three tree plots represent data from Week 0, 1, and 8.

Relative abundances of taxa were compared between responders and non-responders at different phylogenetic levels (Figure 2). Almost no differences between responders and non-responders were observed pre-FMT at W0. However, post-FMT, relative abundances of a number of families were significantly different between outcomes, with responders being enriched for Lachnospiraceae (46.3% [responders, W1, median] *vs*. 22.4% [non-responders, W1, median], *P* = .044; 46.0% [responders, W8] *vs*. 28.5% [non-responders, W8], *P* = .021) and Ruminococcaceae (10.8% [responders, W8] *vs*. 5.6% [non-responders, W8], *P* = .049), while Enterobacteriaceae (0.9% [responders, W1] *vs*. 31.0% [non-responders, W1]; *P* = .003; 0.3% [responders, W8] *vs*. 5.2% [non-responders, W8]; *P* = .017) were more abundant in non-responders.

Bray-Curtis distance at family level compared between outcomes at each time point (**Figure S3**), revealed that responders and non-responders did not differ in their fecal microbiota composition before treatment at W0 (*P* = .329, responders *vs*. non-responders), but differed at W1 (*P* = .025), W8 (*P* = .007) and W26 (*P* = .051), which is in line with the observations of discriminant families (Figure 2). The biplot ordinations of both samples and families consistently showed that Lachnospiraceae and Ruminococcaceae were more abundant in responders, and Enterobacteriaceae were more enriched in non-responders (**Figure S3**). Also the microbial functional potential was observed to be different based on the PICRUSt2 pipeline^18^ (**Figure S4**), suggesting differences in the MetaCyc pathways^19^ for chorismate biosynthesis and aromatic amino acid biosynthesis (**Figure S5**).

Assessment of the change in microbial composition within each outcome before and after FMT (**Figure S6**) revealed that compared to their W0, patients who responded to the treatment had increased abundances of families Ruminococcaceae, Prevotellaceae, Coriobacteriaceae, Porphyromonad-aceae, Bacteroidaceae, Bifidobacteriaceae, and Eubacteriaceae, and reduced abundance of Enterobacteriaceae, Veillonellaceae, Enterococcaceae, and Peptostreptococcaceae. Notably, in non-responders, no families or genera were identified with consistently different abundances before and after FMT.

### Microbial markers for potential prediction of outcome

To identify the taxa at W1 that were potentially indicative of treatment outcome at W8, we first fitted a random forest model for W1 samples at genus level to predict W8 outcomes. The model obtained an area under the curve (AUC) of 0.79 and a prediction accuracy of 73.2% (**Figure S7b**). We summed the importance score of the top 30 most important genera and grouped them at family level (**Figure S7a**). Consistent with the observations in Figure 2, Enterobacteriaceae was the family comprising the most important genera for differentiating outcomes, followed by Lachnospiraceae and Ruminococcaceae.

Next, we identified the two most important and abundant genera (**Figure S8**) from Enterobacteriaceae and Lachnospiraceae, which were *Escherichia* (the most important genus in Enterobacteriaceae, negatively correlated with response) and *Blautia* (the most important genus in Lachnospiraceae, positively correlated with response). The importance of *Escherichia* and *Blautia* was also evidenced by the logistic regression that among all assessed genera (n = 224), only three genera had a significant effect on outcomes (*P* < .05) and *Escherichia* and *Blautia* were the two having the smallest *P* values (*P* = .037 [*Escherichia], P* = .025 [*Blautia*]).

We thus propose that the abundance ratio between *Escherichia* and *Blautia* one week after FMT may function as an indicator of the proceeding response, where a larger value is potentially indicative of non-response to FMTv. We additionally assessed the *Escherichia* to *Blautia* abundance ratio with qPCR and found a good consistency with 16S rRNA gene sequencing (**Figure S9**). Based on this abundance ratio as measured by qPCR, samples could be divided into two groups largely reflecting their treatment outcomes (**Figure S10**).

### Engraftment of donor microbes and improvement of health status

**Figure 3.**
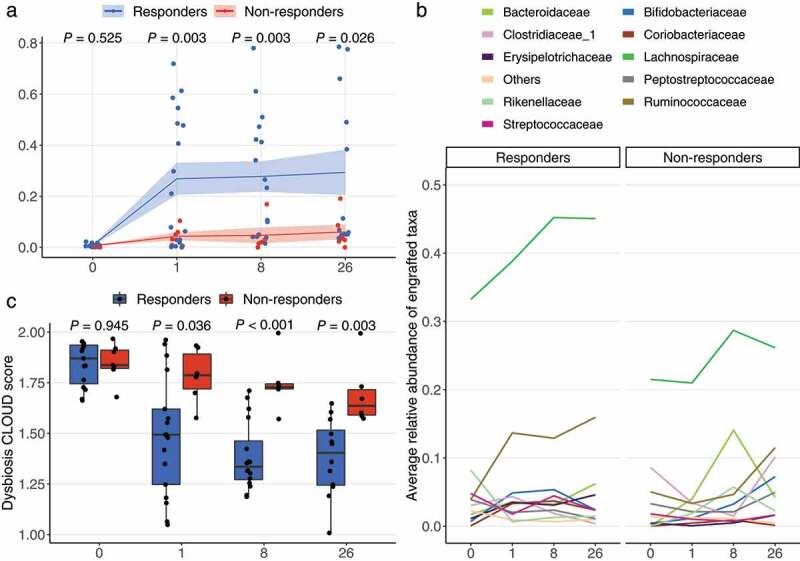
Engraftment of donor microbes and improvement of dysbiosis score. (a) Engraftment of donor microbes was estimated with the SourceTracker program using each patient as the “sink” and the corresponding donor as the “source”. Lines crossing time points represent the mean similarity in the microbial communities between donors and patients. The shaded area indicates standard error of the mean. *P* values were obtained by Welch’s t-test. (b) The top 10 most engrafted families. ASVs engrafted in patients were identified with SourceTracker and their relative abundances were summed at the family level. The top 10 most abundant families are shown and the remaining are merged as “Others”. (c) Dysbiotic status of patients as estimated with the CLOUD test. Patients (test sample) were tested against the donors (reference) using CLOUD, and the derived statistic from the test was used as the dysbiosis score, where smaller scores correspond to healthier bacterial communities. The differences in dysbiosis scores between outcomes were compared with Wilcoxon rank-sum test.

The extent of donor microbiota engraftment in the patient community was investigated by the SourceTracker program^20^ at ASV level, using the patient as the “sink” and the donor sample used for this particular patient as the “source”, to determine the similarity between the patient and the corresponding donor (Figure 3(a)). We found that responders had comparable similarity with non-responders at baseline (0.78% ± 0.7% [W0, responders, mean ± SD] *vs*. 0.62% ± 0.41% [W0, non-responders, mean ± SD]; *P* = .525), but significantly higher similarity at later time points (26.9% ± 26.0% [W1, responders] *vs*. 4.4% ± 3.7% [W1, non-responders], *P* = .003; 27.7% ± 23.8% [W8, responders] *vs*. 4.7% ± 6.9% [W8, non-responders], *P* = .003; 26.3% ± 30.7% [W26, responders] *vs*. 6.1% ± 7.0% [W26, non-responders], *P* = .026).

By use of SourceTracker, we also identified the engrafted ASVs, shown at the family level for the top 10 most abundant (Figure 3(b)), and found that for responders the engrafted ASVs predominantly belonged to the families Lachnospiraceae (33.2% [W0, mean], 38.8% [W1], 45.2% [W8], and 45.1% [W26]) and Ruminococcaceae (3.9% [W0], 13.7% [W1], 12.9% [W8], and 15.9% [W26]). For non-responders, the engrafted ASVs were mainly Lachnospiraceae (21.5% [W0], 21.0% [W1], 28.7% [W8], and 26.1% [W26]) and Bacteroidaceae (0% [W0], 4.0% [W1], 14.0% [W8], and 4.2% [W26]), while no engraftment in non-responders was observed for Ruminococcaceae during the first 8 weeks (5.0% [W0], 3.3% [W1], 4.6% [W8], 11.5% [W26]).

To test whether engraftment of donor microbiota in patients was associated with improvements in gut microbiota health status, we applied a robust non-parametric outlier detection test, designated as CLOUD,^21^ to determine to what extent a CDI patient’s fecal microbiota was different from that of the healthy donors^22^ (Figure 3(c)). Donor samples were used as the reference and the patient samples as test samples. We used the CLOUD statistic (patient sample’s outlier detection test, the smaller value the healthier) as the dysbiosis score and compared these scores between outcomes. Responders and non-responders were shown to have similar dysbiosis scores at W0 (1.870 ± 0.191 [responders] *vs*. 1.837 ± 0.090 [non-responders], *P* = .945, median ± IQR), but thereafter responders had significantly lower dysbiosis scores than non-responders at W1 (1.493 ± 0.373 [responders] *vs*. 1.787 ± 0.1672 [non-responders], *P* = .036), W8 (1.335 ± 0.191 [responders] *vs*. 1.728 ± 0.025 [non-responders], *P* < .001) and W26 (1.404 ± 0.272 [responders] *vs*. 1.636 ± 0.125 [non-responders], *P* = .003).

### The treatment effects of vancomycin and fidaxomicin

**Figure 4.**
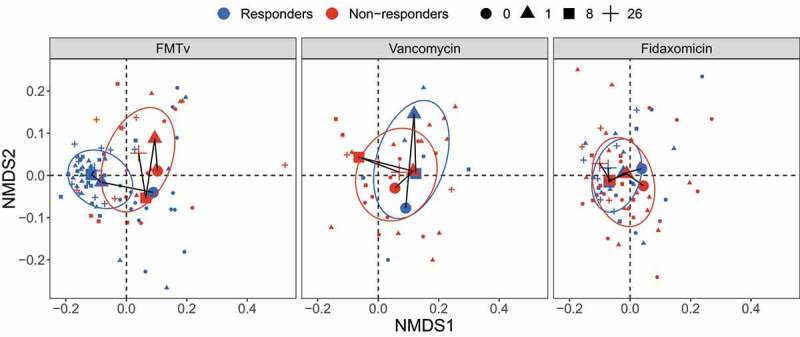
Beta diversity of fecal microbiota for FMTv, vancomycin, and fidaxomicin treatments. Beta diversity is assessed based on the Bray-Curtis distance and visualized by NMDS ordination. Colors of dots and ellipses (50% confidence regions for clusters) refer to different outcomes. Samples are shown with small shapes referring to different time points. Big shapes represent the centroids of sample groups in each condition and are connected with lines according to sample time.

As previously reported, FMTv resulted in a cure rate, defined as clinical resolution and negative PCR test for *C. difficile*, of 71%. This was significantly higher than observed for fidaxomicin (33%) and vancomycin (19%).^17^ Here, we investigated the microbiota of patients randomized to vancomycin (n = 16) and fidaxomicin (n = 24) (**Figure S1**). To assess whether these two antibiotic treatments led to similar microbial changes in responders and non-responders as FMTv, we compared their beta diversity based on Bray-Curtis distances and visualized with the nonmetric multidimensional scaling (NMDS) ordination (Figure 4). Consistent with the previous visualization in Figure 1(c), the responders and non-responders in FMTv showed distinct trajectories over time. In contrast, analysis of samples from vancomycin and fidaxomicin treated patients did not show clear differences in the microbiota between outcomes, which is consistent with the permutational multivariate analysis of variance (PERMANOVA) (**Table S2**). We additionally observed that fidaxomicin led to increased richness over time in the responders, but slower and to a less extent than observed in the FMTv treated patients (**Figure S11**).

## Discussion

We followed the gut microbiota changes in patients with multiple, recurrent CDI treated with FMTv, and compared the gut microbiota differences between outcomes as determined at W8. Before FMT (W0), no significant differences were observed between responders and non-responders to this treatment. However, already 1 week after FMT, substantial differences in the gut microbiota were found between outcomes. Similar microbial differences were not observed in patients treated with antibiotics monotherapy, participating in the same randomized clinical study.

One week after FMTv treatment, responders and non-responders differed in their gut microbiota in the following ways: (i) Alpha diversity (observed richness and Shannon diversity) increased significantly in responders compared to pre-FMT, but remained unchanged in patients that did not respond to FMT. (ii) Beta diversity analysis revealed that responders, but not non-responders, had a different gut microbiota composition than before the FMT. Also, responders were more similar to healthy donors in their gut microbiota than non-responders. (iii) Responders were found to be depleted for Enterobacteriaceae and enriched for Lachnospiraceae and Ruminococcaceae. (iv) Engraftment of donor microbes in responders was significantly more prevalent than in non-responders. (v) Responders had a more pronounced resolution of microbiota dysbiosis than non-responders.

Our findings of significant microbial differences between outcomes as early as 1 week after FMT are in line with two previous reports,^15,16^ supporting that fecal microbiota patterns that differentiate between outcomes, indeed occur early after FMT. While the previous reports relate to FMT given as ingested capsules, the present study applied colonoscopy or a nasojejunal tube for the treatment, suggesting that the changes occur independently of the application method. The inclusion of comparator groups and the application of a randomized design in the current trial allowed us to clearly establish the predictive value of microbiota changes.

Compared to non-responders, patients who responded to FMTv or FMT were distinguished by higher abundances of Lachnospiraceae and Ruminococcaceae, and lower abundance of Enterobacteriaceae. A high abundance of Lachnospiraceae and Ruminococcaceae is suggested as a feature of super-donors.^9^ Members of these families including the short-chain-fatty acid producing genera *Butyricicoccus*,^23^ *Roseburia*,^24^ *Blautia*, and *Dorea*^25^ were significantly more abundant in responders and in line with our findings, are reported to be associated with prevention of recurrence.^15^ Also, a study in mice shows that the precolonization of isolates from the Lachnospiraceae family suppresses the growth of *C. difficile*.^26^ Enterobacteriaceae on the other hand are reported to be positively correlated with recurrence.^12,15^ Also, accompanied by the changes in taxonomic composition, the functional potential predicted with PICRUSt2 was different between responders and non-responders. Lachnospiraceae and Ruminococcaceae were positively associated with a number of pathways, such as the Chorismate biosynthesis and aromatic amino acid biosynthesis that were previously reported to be protective against CDI,^27^ while Enterobacteriaceae were negatively correlated with these pathways. The distinct abundance characteristics of Lachnospiraceae and Ruminococcaceae between outcomes early after FMT were likely due to the differences in the engrafted donor microbes, where Lachnospiraceae and Ruminococcaceae engrafted substantially in responders one week post-FMT, but not in non-responders. Overall, members of these taxa potentially represent indicative microbial markers that can be used for outcome prediction.

Previous studies have predicted outcomes based on complex regression tree analyses.^15^ Here, we proposed a way to construct simpler outcome indexes based on the identified microbial markers. In the present study, the abundance ratio of two genera, namely *Escherichia* and *Blautia* measured at Week 1, predicted the clinical outcomes at Week 8 with very good performance (AUC = 0.92). The strength of this method relies on the facts that *Escherichia* and *Blautia* are quite abundant genera in both responders and non-responders, which facilitates quantification, and that qPCR is a well-established technique in the clinic. The associations of the given genera with outcomes are consistent with observations reported by others.^16^ Our results suggest that future studies regarding outcome predictions may benefit from using a limited number of taxa, measurable by clinically feasible methods. However, we must pinpoint that due to the existing variations in different cohorts because of study design, population size, individual heterogeneity, *etc*., a general outcome index or prediction method needs to be thoroughly validated.

The effects on the microbiota observed after FMTv were very different from those observed after treatment with the antibiotics vancomycin and fidaxomicin monotherapies, and no difference between responders and non-responders was detected after antibiotic treatment. Vancomycin and fidaxomicin did not lead to recovery of gut microbiota structure as seen for FMTv in the group of responders, although a delayed and weak increase in richness occurred at W8 and W26 in the vancomycin group. Our observations underpin that while fidaxomicin and vancomycin inhibit the immediate growth of *C. difficile*,^28^ FMT induces a sustained treatment response through more complicated and probably more robust mechanisms.^29^

## Conclusions

We characterized the substantial differences in the gut microbiota composition between responders and non-responders to FMT, which occurred already one week after FMT against recurrent *C. difficile* infection. Based on the observed differences, we propose a way to construct predictive outcome indexes based on simple and clinically feasible methods. Additionally, we found that the gut microbiota differences between outcomes after treatment with vancomycin or fidaxomicin monotherapies did not resemble the differences observed after FMT, highlighting that FMT leads to treatment response in a different way than antibiotics.

## Materials and methods

### Study design and clinical outcomes

We analyzed fecal samples from our previously published randomized clinical trial,^17^ which compared three different treatment strategies for recurrent CDI, namely FMT preceded by vancomycin (FMTv), vancomycin, and fidaxomicin. Of 120 patients screened in the study, 64 patients in total were randomized to either FMTv (n = 24), vancomycin (n = 16), or fidaxomicin (n = 24) (**Figure S1**). In brief, criteria for inclusion were minimum 18 years, at least 3 liquid stools per day, a positive polymerase chain reaction (PCR) test result for CD toxin A, toxin B, or binary toxin, and at least 1 prior treatment course with vancomycin or fidaxomicin for CDI. Exclusion criteria were pregnancy or breastfeeding, any ongoing antibiotic treatment, and use of drugs with a known interaction with vancomycin or fidaxomicin. Eventually, 64 patients with a median age of 68 years (range 21–92 years) and a median of 4 (range 2–10 episodes) previous CDI episodes were included in the study.^17^

In the FMTv group, the 24 patients were treated with 4–10 days of vancomycin 125 mg 4 times daily, followed by FMT. Fecal samples from this group were investigated to identify the microbial differences between outcomes and whether these may be used as biomarkers to predict treatment effect. Additionally, samples from the 16 patients allocated to vancomycin (10 days, 125 mg 4 times daily) and 24 patients allocated to fidaxomicin (10 days, 200 mg twice daily) were investigated.

Global, i.e. combined clinical and microbiological, outcomes were determined 8 weeks after FMT or antibiotic treatments, and were based on combined clinical resolution and a negative PCR test for *C. difficile* carried out as previously described.^17^ This study evaluated the effect following a single FMT.

All patients provided written informed consent before inclusion. The study protocol was approved by the Central Denmark Region Ethics Committee (j.no. 1–10-72-2577-15) and by the Danish Medicines Agency (j.no. 2015092214). Data storage was approved by the Danish Data Protection Agency (j.no. 1–16-02-15-16). The study was conducted according to the principles for Good Clinical Practice and was monitored by the Good Clinical Practice Unit at the Aarhus and Aalborg University Hospitals (j. no. 2015/589).

### Fecal microbiota transplantation and fecal samples

Healthy stool donors were recruited and screened at the public blood bank at Aarhus University Hospital (Aarhus, Denmark) as previously reported.^30^ FMT was performed either by colonoscopy using 50 g of frozen-thawed donor feces preparation (77.6%), or by nasojejunal tube (22.4%) when colonoscopy was not possible. Processing of the donor material and clinical procedures are described previously.^17,31^

A total of 24 processed fecal samples were from the six healthy donors who provided feces for the FMTs. For patients allocated to FMTv, a total of 85 fecal samples were collected before vancomycin treatment and FMT (W0), and 1-week (W1), 8-weeks (W8) and 26-weeks (W26) after FMT. Fecal samples were snap-frozen in the patient’s home at −20°C and transferred to the lab where they were stored at −80°C until analyses. For patients allocated to vancomycin and fidaxomicin, a total of 98 fecal samples were collected in the same way as for FMTv.

### DNA extraction, sequencing, and bioinformatics analysis

Microbial DNA was extracted using 0.18–0.22 g of feces content of each sample with the DNeasy® PowerLyzer® PowerSoil® isolation kit from Qiagen. The extracted DNA was stored at −20°C before use. The V3 region of 16S rRNA gene was amplified with PhusionTM High-Fidelity (HF) DNA Polymerase kit from Thermo Fisher Scientific and primers 341 F (5’-CCTACGGGAGGCAGCAG-3’) and 518 R (5’-ATTACCGCGGCTGCTGG-3’), which were modified from a previous report.^32^ Amplified PCR products were purified with HighPrep^TM^ PCR Clean-up System, the DNA concentration was determined and normalized with Qubit® 2.0 Flourmeter, and up to 96 amplified products were pooled as a library. Single-end sequencing was performed with the Ion Torrent S5^TM^ platform with an Ion 520™ Chip. The raw FASTQ files were demultiplexed with QIIME2,^33^ adapter and sequencing primer were removed with cutadapt (version 2.10),^34^ and the final sequence length was trimmed to range from 125 bp to 180 bp. The pre-processed reads were analyzed with the DADA2 pipeline in R,^35^ using parameters recommended for Ion Torrent and with “pool” set as “TRUE”, and other parameters set as default. With the DADA2 algorithm, taxonomic assignments were resolved with exact sequence features, called amplicon sequence variants (ASVs). Taxonomy was assigned using the Ribosomal Database Project (RDP) database (release 11.5).^36^

### Quantitative PCR

Relative quantification of specific genera was performed using primers targeting *Escherichia* spp. (forward: CATTGACGTTACCCGCAGAAGAAGC, reverse: CTCTACGAGACTCAAGCTTGC)^37^ and *Blautia* spp. (forward: GTGAAGGAAGAAGTATCTCGG, reverse: TTGGTAAGGTTCTTCGCGTT),^38^ in combination with universal bacterial primers (forward: ACTCCTACGGGAGGCAGCAGT and reverse: GTATTACCGCGGCTGCTGGCAC)^39^ using the Δ-Ct method. The genera specific primers were validated *in silico* in the RDP web tool^36^ and found to have 100% identity match with 7/8 members of the *Escherichia* genus and 8/9 members of the *Blautia* genus when searching good quality type strains ≥ 1200 bp and only a single mismatched species within the Gamma-proteobacteria (*Erwinia* spp.). The PCR reactions each contained 5.5 µl LightCycler® 480 II SYBR Green I Master (Roche Diagnostics A/S, Hvidovre, Denmark), 0.2 µM of each primer and 2 µl template DNA in a total reaction volume of 11 µl. Reactions conditions were as follows: Initial 95°C for 5 minutes, followed by 45 cycles of 95°C for 10 seconds, 60°C for 15 seconds and 72°C for 45 seconds. Finally, a melting curve was generated by gradually increasing the temperature (95°C for 5 seconds, 68°C for 1 minute and increasing the temperature to 98°C with a rate of 0.11°C/second with continuous fluorescence detection). The qPCR was performed in triplicate for each sample/primer combination in 384-well plate format on a LightCycler® 480 II instrument (Roche Applied Science) and analyzed using the dedicated LightCycler® 480 software using the fit-point method.

### Statistical analysis

Samples were rarefied at 10,000 reads before calculating the alpha diversity with the R-package “phyloseq” (**Figure S12**).^40^ The difference in the median and mean alpha diversity between groups were performed with Wilcoxon rank-sum test and Welch’s t-test, respectively. Beta diversity was estimated with Bray-Curtis distance matrix and tested with permutational multivariate analysis of variance (PERMANOVA, “adonis”, R-package “vegan”).^41^ The functional potential of the fecal microbiota was predicted with phylogenetic investigation of communities by reconstruction of unobserved states (PICRUSt2) and identified as metabolic pathways (MetaCyc).^18,19^ Correlations between pathways and taxa abundances were determined using Spearman’s rank-order correlation coefficient (function “rcorr” in R-package “Hmisc”) and were plotted with the R-package “corrplot”. The taxonomic composition comparison between outcomes was tested with Wilcoxon rank-sum test, or within outcome but between time points with paired Wilcoxon rank-sum test. The engraftment of the donor microbiota was determined by the SourceTracker program with default parameters as the percentage of ASVs in a patient’s fecal sample (sink) that was attributed to donor microbiota (source).^20^ The random forest model was built at the genus level with the function “imbalanced” (ntree = 10,000, method = “rqf”, importantance = “TRUE”, splitrule = “auc”; all other parameters are in default) in the R-package “randomForestSRC” to address the imbalanced sample size between outcomes.^42^ The prediction accuracy, AUC (area under the receiver operating characteristic (ROC) curve) value and variable importance from the random forest model were obtained from the 10 cycles of 10-fold cross-validation (100 iterations in total). The logistic regression was performed with the function “glm” in R-package “stats”.

The optimal threshold of abundance ratio to differentiate responders from non-responders was determined by the Youden’s J statistic (R-package “OptimalCutpoints”).^43^ The binary outcomes were predicted based on this threshold. AUC was used to measure the discriminatory power of the model. The health status of gut microbiota was determined by the CLOUD test (k.neighbor is the entire sample size of donors),^21^ and the statistic (outlier detection test) from the CLOUD test was used as the dysbiosis score. We applied the Benjamini-Hochberg *P* values correction to account for the false discovery rate and the adjusted *P* values were shown as “*P_adj_*” (“p.adjust” in R-package “stats”).^44^ Statistical significance level was set at 0.05. The taxonomic composition shown with trees was generated with R-package “metacoder”.^45^ Most plots were generated with R-package “ggplot2”.^46^

## Supplementary Material

Supplemental Material

## Data Availability

The raw sequence data of 16S rRNA gene sequencing reported in this paper are available from the National Center for Biotechnology Information (NCBI) with the Sequence Read Archive (SRA) bioproject number PRJNA797470. All other data and the code used to reanalyze the data reported in this paper is available from the corresponding author upon request. ncbi.nlm.nih.gov/sra/?term=PRJNA797470

## References

[1] Khanna S, Pardi DS, Aronson SL, Kammer PP, Orenstein R, St Sauver JL, Harmsen WS, Zinsmeister AR. The epidemiology of community-acquired clostridium difficile infection: a population-based study. Am J Gastroenterol. 2012;107(1):89–13. doi:10.1038/ajg.2011.398. 22108454 PMC3273904

[2] McDonald LC, Gerding DN, Johnson S, Bakken JS, Carroll KC, Coffin SE, Dubberke ER, Garey KW, Gould CV, Kelly C, et al. Clinical practice guidelines for clostridium difficile infection in adults and children: 2017 update by the infectious diseases society of America (IDSA) and society for healthcare epidemiology of America (SHEA). Clin Infect Dis. 2018;66(7):e1–48. doi:10.1093/cid/cix1085. 29462280 PMC6018983

[3] van Prehn J, Reigadas E, Vogelzang EH, Bouza E, Hristea A, Guery B, Krutova M, Norén T, Allerberger F, Coia JE, et al. European society of clinical microbiology and infectious diseases: 2021 update on the treatment guidance document for clostridioides difficile infection in adults. Clin Microbiol Infect [Internet]. 2021 accessed 2022 Jan 21;27(2):S1–21. https://pubmed.ncbi.nlm.nih.gov/34678515/10.1016/j.cmi.2021.09.038 34678515

[4] Leffler DA, Lamont JT, Longo DL. Clostridium difficile infection. N Engl J Med. 2015;372(16):1539–1548. doi:10.1056/NEJMra1403772. 25875259

[5] Lai CY, Sung J, Cheng F, Tang W, Wong SH, Chan PKS, Kamm MA, Sung JJY, Kaplan G, Chan FKL, et al. Systematic review with meta-analysis: review of donor features, procedures and outcomes in 168 clinical studies of faecal microbiota transplantation. Aliment Pharmacol Ther. 2019;49(4):354–363. doi:10.1111/apt.15116. 30628108

[6] Gough E, Shaikh H, Manges AR. Systematic review of intestinal microbiota transplantation (fecal bacteriotherapy) for recurrent clostridium difficile infection. Clin Infect Dis. 2011;53(10):994–1002. doi:10.1093/cid/cir632. 22002980

[7] Baunwall SMD, Lee MM, Eriksen MK, Mullish BH, Marchesi JR, Dahlerup JF, Hvas CL. Faecal microbiota transplantation for recurrent Clostridioides difficile infection: an updated systematic review and meta-analysis. EClinicalMedicine. 2020;29–30:100642. doi:10.1016/j.eclinm.2020.100642. PMC7788438 33437951

[8] Aroniadis OC, Brandt LJ. Fecal microbiota transplantation: past, present and future. Curr Opin Gastroenterol. 2013;29(1):79–84. doi:10.1097/MOG.0b013e32835a4b3e. 23041678

[9] Wilson BC, Vatanen T, Cutfield WS, O’Sullivan JM. The super-donor phenomenon in fecal microbiota transplantation. Front Cell Infect Microbiol. 2019;9:1–11. doi:10.3389/fcimb.2019.00002. 30719428 PMC6348388

[10] Khanna S, Vazquez-Baeza Y, González A, Weiss S, Schmidt B, Muñiz-Pedrogo DA, Rainey JF, Kammer P, Nelson H, Sadowsky M, et al. Changes in microbial ecology after fecal microbiota transplantation for recurrent C. difficile infection affected by underlying inflammatory bowel disease. Microbiome. 2017;5(1):55. doi:10.1186/s40168-017-0269-3. 28506317 PMC5433077

[11] Staley C, Kelly CR, Brandt LJ, Khoruts A, Sadowsky MJ, Blaser MJ. Complete microbiota engraftment is not essential for recovery from recurrent Clostridium difficile infection following fecal microbiota transplantation. MBio. 2016;7(6):1–9. doi:10.1128/mBio.01965-16. PMC5181777 27999162

[12] Weingarden A, González A, Vázquez-Baeza Y, Weiss S, Humphry G, Berg-Lyons D, Knights D, Unno T, Bobr A, Kang J, et al. Dynamic changes in short- and long-term bacterial composition following fecal microbiota transplantation for recurrent Clostridium difficile infection. Microbiome. 2015;3(1):1–8. doi:10.1186/s40168-015-0070-0. 25825673 PMC4378022

[13] Cammarota G, Ianiro G, Tilg H, Rajilić-Stojanović M, Kump P, Satokari R, Sokol H, Arkkila P, Pintus C, Hart A, et al. European consensus conference on faecal microbiota transplantation in clinical practice. Gut. 2017;66(4):569–580. doi:10.1136/gutjnl-2016-313017. 28087657 PMC5529972

[14] Mullish BH, Quraishi MN, Segal JP, McCune VL, Baxter M, Marsden GL, Moore DJ, Colville A, Bhala N, Iqbal TH, et al. The use of faecal microbiota transplant as treatment for recurrent or refractory Clostridium difficile infection and other potential indications: joint british society of gastroenterology (BSG) and healthcare infection society (HIS) guidelines. Gut. 2018;67(11):1920–1941. doi:10.1136/gutjnl-2018-316818. 30154172

[15] Staley C, Kaiser T, Vaughn BP, Graiziger CT, Hamilton MJ, Rehman TU, Song K, Khoruts A, Sadowsky MJ. Predicting recurrence of Clostridium difficile infection following encapsulated fecal microbiota transplantation. Microbiome. 2018;6(1):1–13. doi:10.1186/s40168-018-0549-6. 30227892 PMC6145197

[16] Staley C, Vaughn BP, Graiziger CT, Singroy S, Hamilton MJ, Yao D, Chen C, Khoruts A, Sadowsky MJ. Community dynamics drive punctuated engraftment of the fecal microbiome following transplantation using freeze-dried, encapsulated fecal microbiota. Gut Microbes. 2017;8(3):276–288. doi:10.1080/19490976.2017.1299310. 28282270 PMC5479395

[17] Hvas CL, Dahl Jørgensen SM, Jørgensen SP, Storgaard M, Lemming L, Hansen MM, Erikstrup C, Dahlerup JF. Fecal microbiota transplantation is superior to fidaxomicin for treatment of recurrent clostridium difficile infection. Gastroenterol. 2019;156(5):1324–1332.e3. doi:10.1053/j.gastro.2018.12.019. 30610862

[18] Douglas GM, Maffei VJ, Zaneveld JR, Yurgel SN, Brown JR, Taylor CM, Huttenhower C, Langille MGI. PICRUSt2 for prediction of metagenome functions. Nat Biotechnol. 2020;38(6):685–688. doi:10.1038/s41587-020-0548-6. 32483366 PMC7365738

[19] Caspi R, Billington R, Keseler IM, Kothari A, Krummenacker M, Midford PE, Ong WK, Paley S, Subhraveti P, Karp PD. The MetaCyc database of metabolic pathways and enzymes-a 2019 update. Nucleic Acids Res. 2020;48(D1):D445–53. doi:10.1093/nar/gkz862. 31586394 PMC6943030

[20] Knights D, Kuczynski J, Charlson ES, Zaneveld J, Mozer MC, Collman RG, Bushman FD, Knight R, Kelley ST. Bayesian community-wide culture-independent microbial source tracking. Nat Methods. 2011;8(9):761–765. doi:10.1038/nmeth.1650. 21765408 PMC3791591

[21] Montassier E, Al-Ghalith GA, Hillmann B, Viskocil K, Kabage AJ, McKinlay CE, Sadowsky MJ, Khoruts A, Knights D. CLOUD: a non-parametric detection test for microbiome outliers. Microbiome. 2018;6(1):137. doi:10.1186/s40168-018-0514-4. 30081949 PMC6080375

[22] Wei S, Bahl MI, Baunwall SMD, Hvas CL, Licht TR, Drake HL. Determining gut microbial dysbiosis: a review of applied indexes for assessment of intestinal microbiota imbalances. Appl Environ Microbiol. 2021;87(11):1–13. doi:10.1128/AEM.00395-21. PMC8208139 33741632

[23] Li X, Chu Q, Huang Y, Xiao Y, Song L, Zhu S, Kang Y, Lu S, Xu J, Ren Z. Consortium of probiotics attenuates colonization of clostridioides difficile. Front Microbiol. 2019;10:1–12. doi:10.3389/fmicb.2019.02871. 31921049 PMC6920126

[24] La Rosa SL, Leth ML, Michalak L, Hansen ME, Pudlo NA, Glowacki R, Pereira G, Workman CT, Arntzen M, Pope PB, et al. The human gut firmicute roseburia intestinalis is a primary degrader of dietary β-mannans. Nat Commun. 2019;10(1):1–14. doi:10.1038/s41467-019-08812-y. 30796211 PMC6385246

[25] Rodríguez C, Romero E, Garrido-Sanchez L, Alcaín-Martínez G, Andrade RJ, Taminiau B, Daube G, García-Fuentes E. Microbiota insights in clostridium difficile infection and inflammatory bowel disease. Gut Microbes. 2020;12(1):1–25. doi:10.1080/19490976.2020.1725220. PMC7524151 32129694

[26] Reeves AE, Koenigsknecht MJ, Bergin IL, Young VB, McCormick BA. Suppression of Clostridium difficile in the gastrointestinal tracts of germfree mice inoculated with a murine isolate from the family lachnospiraceae. Infect Immun. 2012;80(11):3786–3794. doi:10.1128/IAI.00647-12. 22890996 PMC3486043

[27] Pérez-Cobas AE, Artacho A, Ott SJ, Moya A, Gosalbes MJ, Latorre A, Rhee JH. Structural and functional changes in the gut microbiota associated to Clostridium difficile infection. Front Microbiol. 2014;5:1–15. doi:10.3389/fmicb.2014.00001. 25309515 PMC4163665

[28] Allen CA, Babakhani F, Sears P, Nguyen L, Sorg JA. Both fidaxomicin and vancomycin inhibit outgrowth of Clostridium difficile spores. Antimicrob Agents Chemother. 2013;57(1):664–667. doi:10.1128/AAC.01611-12. 23147724 PMC3535933

[29] Khoruts A, Sadowsky MJ. Understanding the mechanisms of faecal microbiota transplantation. Nat Rev Gastroenterol Hepatol. 2016;13(9):508–516. doi:10.1038/nrgastro.2016.98. 27329806 PMC5909819

[30] Jørgensen SMD, Erikstrup C, Dinh KM, Lemming LE, Dahlerup JF, Hvas CL. Recruitment of feces donors among blood donors: results from an observational cohort study. Gut Microbes. 2018;9(6):540–550. doi:10.1080/19490976.2018.1458179. 29617178 PMC6287698

[31] Jørgensen SMD, Hansen MM, Erikstrup C, Dahlerup JF, Hvas CL. Faecal microbiota transplantation: establishment of a clinical application framework. Eur J Gastroenterol Hepatol. 2017;29(11):e36–45. doi:10.1097/MEG.0000000000000958. 28863010

[32] Milani C, Hevia A, Foroni E, Duranti S, Turroni F, Lugli GA, Sanchez B, Martín R, Gueimonde M, van Sinderen D, et al. Assessing the fecal microbiota: an optimized ion torrent 16S rRNA gene-based analysis protocol. PLoS One [Internet]. 2013;8(7):e68739. [accessed 2014 Jan 25]. http://www.pubmedcentral.nih.gov/articlerender.fcgi?artid=3711900&tool=pmcentrez&rendertype=abstract. 23869230 10.1371/journal.pone.0068739 PMC3711900

[33] Bolyen E, Rideout JR, Dillon MR, Bokulich NA, Abnet CC, Al-Ghalith GA, Alexander H, Alm EJ, Arumugam M, Asnicar F, et al. Reproducible, interactive, scalable and extensible microbiome data science using QIIME 2. Nat Biotechnol. 2019;37(8):852–857. doi:10.1038/s41587-019-0209-9. 31341288 PMC7015180

[34] Martin M. Cutadapt removes adapter sequences from high-throughput sequencing reads. EMBnet.journal. 2011;17(1):10. doi:10.14806/ej.17.1.200.

[35] Callahan BJ, McMurdie PJ, Rosen MJ, Han AW, Johnson AJA, Holmes SP. DADA2: high-resolution sample inference from Illumina amplicon data. Nat Methods. 2016;13(7):581–583. doi:10.1038/nmeth.3869. 27214047 PMC4927377

[36] Cole JR, Wang Q, Cardenas E, Fish J, Chai B, Farris RJ, Kulam-Syed-Mohideen AS, McGarrell DM, Marsh T, Garrity GM, et al. The ribosomal database project: improved alignments and new tools for rRNA analysis. Nucleic Acids Res. 2009;37(Database):141–145. doi:10.1093/nar/gkn879. PMC2686447 19004872

[37] Bartosch S, Fite A, Macfarlane GT, McMurdo MET. Characterization of bacterial communities in feces from healthy elderly volunteers and hospitalized elderly patients by using real-time PCR and effects of antibiotic treatment on the fecal microbiota. Appl Environ Microbiol. 2004;70(6):3575. doi:10.1128/AEM.70.6.3575-3581.2004. 15184159 PMC427772

[38] Kurakawa T, Ogata K, Matsuda K, Tsuji H, Kubota H, Takada T, Kado Y, Asahara T, Takahashi T, Nomoto K. Diversity of intestinal Clostridium coccoides group in the Japanese population, as demonstrated by reverse transcription-quantitative PCR. PLoS One. 2015;10(5):e0126226. doi:10.1371/journal.pone.0126226. 26000453 PMC4441462

[39] Walter J, Tannock GW, Tilsala-Timisjarvi A, Rodtong S, Loach DM, Munro K, Alatossava T. Detection and identification of gastrointestinal lactobacillus species by using denaturing gradient gel electrophoresis and species-specific PCR primers. Appl Environ Microbiol. 2000;66(1):297–303. doi:10.1128/AEM.66.1.297-303.2000. 10618239 PMC91821

[40] McMurdie PJ, Holmes S, Watson M. phyloseq: an R package for reproducible interactive analysis and graphics of microbiome census data. PLoS One. 2013;8(4):e61217. doi:10.1371/journal.pone.0061217. 23630581 PMC3632530

[41] McArdle BH, Anderson MJ. Fitting multivariate models to community data: a comment on distance-based redundancy analysis. Ecology. 2001;82(1):290–297. doi:10.1890/0012-9658(2001)082[0290:FMMTCD]2.0.CO;2.

[42] Ishwaran H, Kogalur UB, Kogalur MUB. Package ‘randomForestSRC’. 2021. https://cran.r-project.org/web/packages/randonForestSRC/index.html.

[43] López-Ratón M, Rodríguez-Álvarez MX, Suárez CC, Sampedro FG. OptimalCutpoints: an R package for selecting optimal cutpoints in diagnostic tests. J Stat Softw. 2014;61(8):1–36. doi:10.18637/jss.v061.i08.

[44] Benjamini Y, Hochberg Y. Controlling the false discovery rate: a practical and powerful approach to multiple testing. J R Stat Soc Ser B. 1995;57:289–300.

[45] Foster ZSL, Sharpton TJ, Grünwald NJ, Poisot T. Metacoder: an R package for visualization and manipulation of community taxonomic diversity data. PLoS Comput Biol. 2017;13(2):e1005404. doi:10.1371/journal.pcbi.1005404. 28222096 PMC5340466

[46] Wickham H. ggplot2. Wiley Interdiscip Rev Comput Stat. 2011;3(2):180–185. doi:10.1002/wics.147.

