# Supplementary material for: Gut microbiota differs between treatment outcomes early after fecal microbiota transplantation against recurrent Clostridioides difficile infection
Source: Gut Microbes. 2022 Jun 5;14(1):2084306. doi: 10.1080/19490976.2022.2084306 (PMC9176232; doi:10.1080/19490976.2022.2084306)
Supplement: Supplemental Material [file KGMI_A_2084306_SM3864.pdf]

Supplementary materials

Gut microbiota differs between treatment outcomes early after fecal microbiota transplantation against recurrent *Clostridioides difficile* infection

Shaodong Wei[1], Martin Iain Bahl[1], Simon Mark Dahl Baunwall[2], Jens Frederik Dahlerup[2], Christian Lodberg Hvas[2], Tine Rask Licht[1,*]

Affiliations:
[1]National Food Institute, Technical University of Denmark, Kemitorvet 202, 2800 Kgs Lyngby, Denmark
[2]Department of Hepatology and Gastroenterology, Aarhus University Hospital, Aarhus, Denmark

[*]For correspondence: trli@food.dtu.dk

Supplementary figures and tables

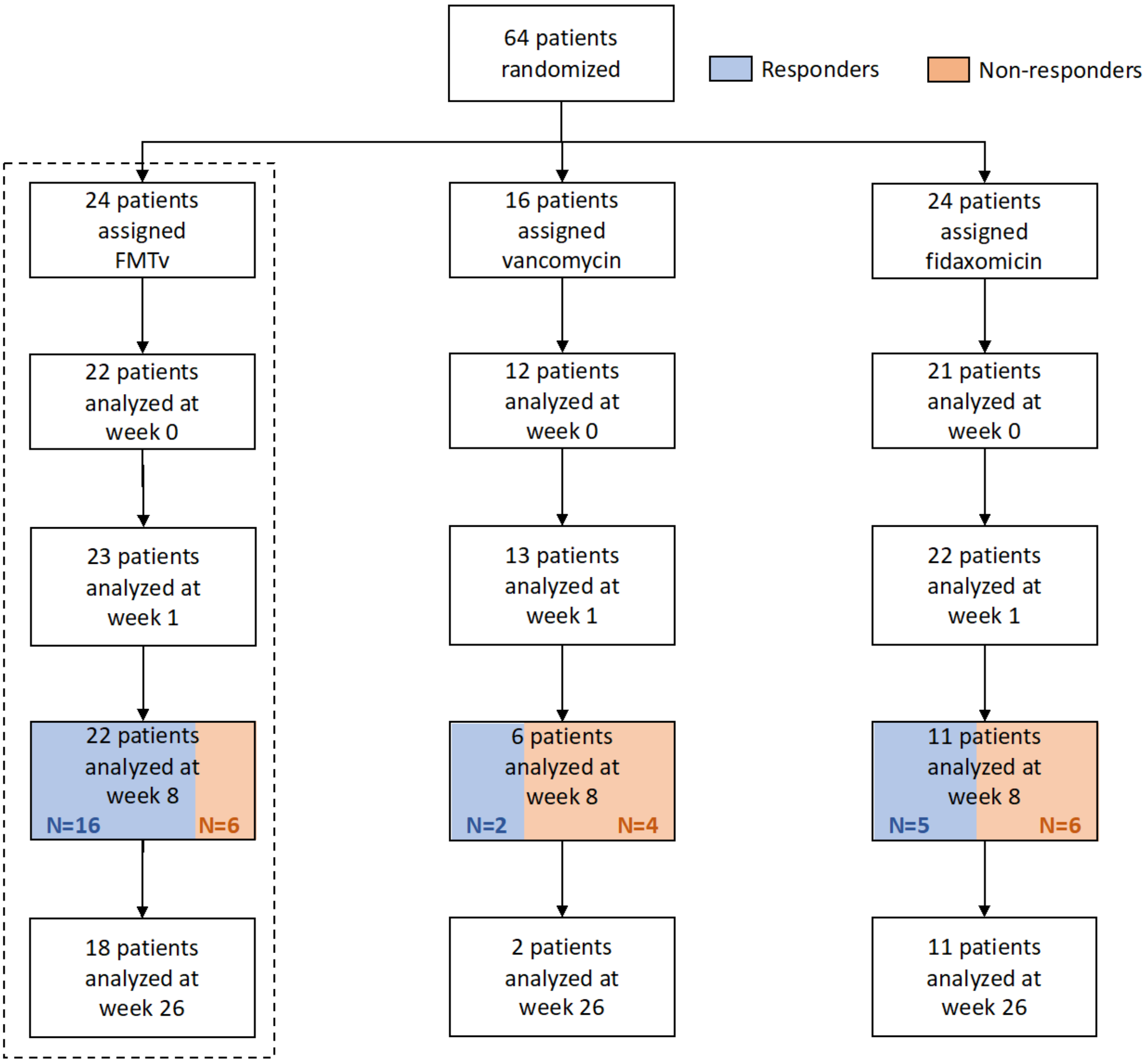

Figure S1. Study design of the clinical trial. Patients were randomly allocated to three treatment arms, namely FMTv (FMT preceded by vancomycin), fidaxomicin, and vancomycin. FMTv group was used to assess the effect of FMT on the gut microbiota and is highlighted with a box in dashed lines. The vancomycin and fidaxomicin groups were used to compare the outcome-associated changes induced by FMT to those induced by antibiotic treatment. Fecal samples were collected before any treatments (FMTv or antibiotics) (W0), and 1 (W1), 8 (W8), and 26 weeks (W26) after treatments. Blue and orange colors refer to responders and non-responders at W8, respectively. Outcomes were determined based on combined clinical resolution and a negative PCR test. The width of blue and orange colors is proportional to the number of patients within each outcome.

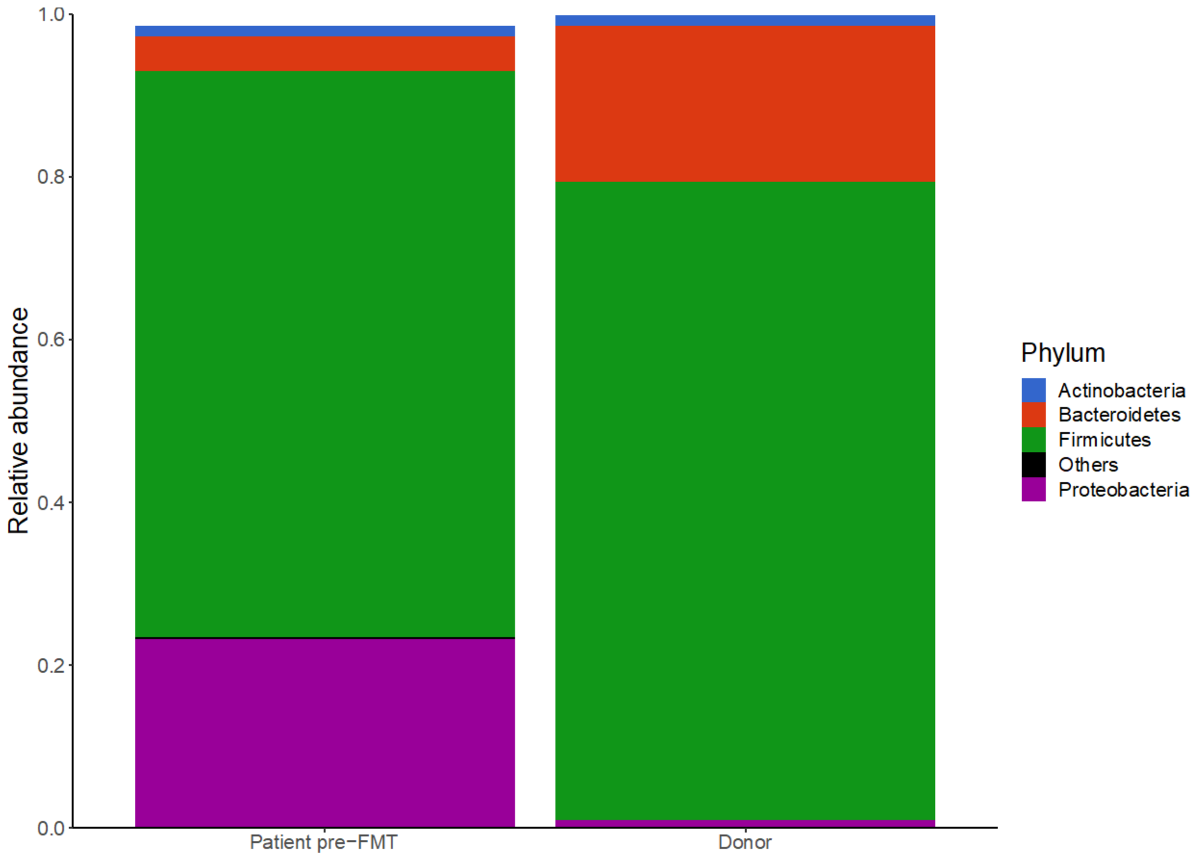

Figure S2. Relative abundance of the top four most abundant phyla in patients pre-FMT and in donors. The four most abundant phyla are colored as indicated, and the less abundant phyla are merged as “Others”.

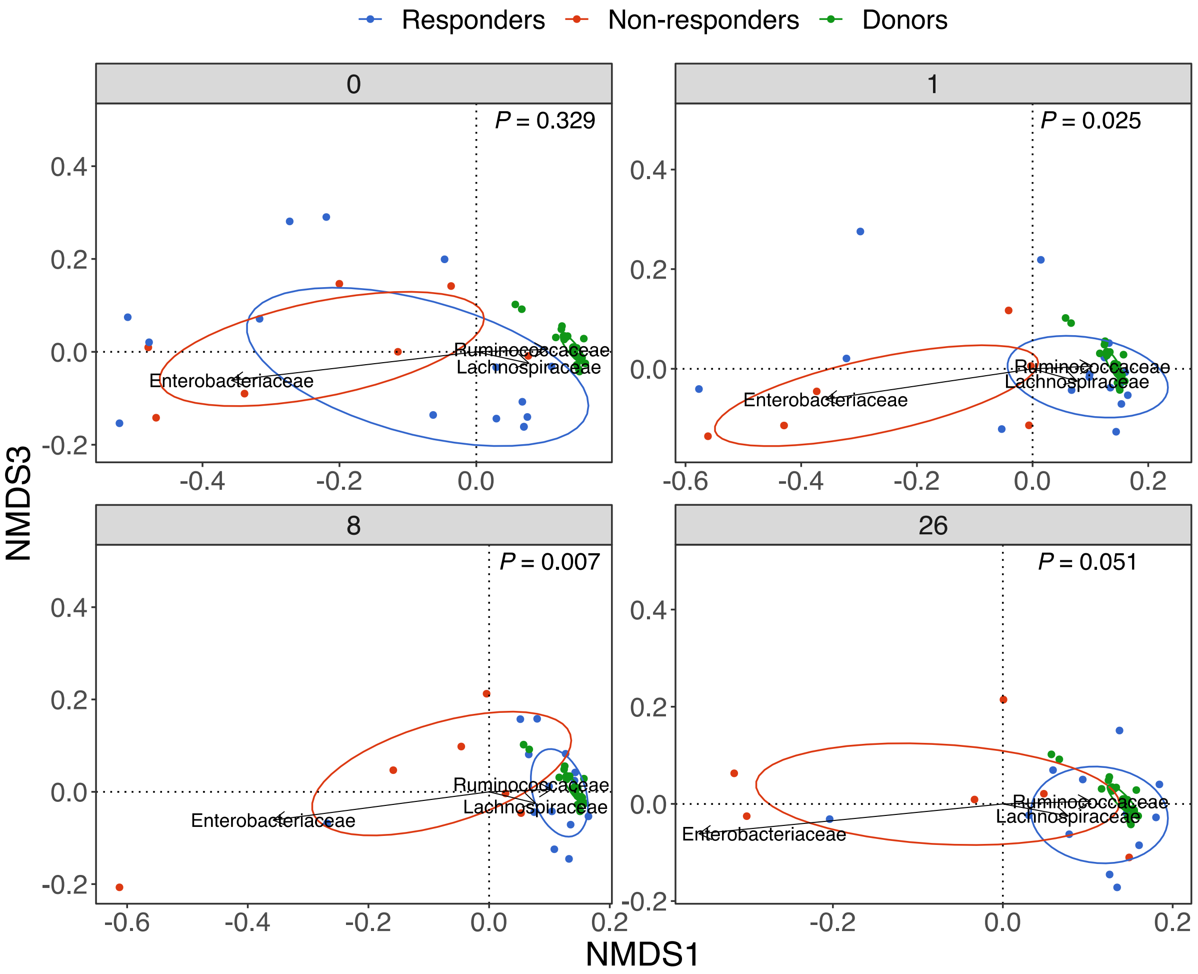

Figure S3. Beta diversity of samples and taxa estimated with Bray-Curtis distance and visualized with nonmetric multidimensional scaling (NMDS) ordination. Bray-Curtis distance was estimated at the family level. Dots are samples colored differently according to their outcomes with ellipses indicating 50% confidence regions for clusters. Arrows point to the families directing the effect and the arrow lengths indicate the strength of the association. Comparison of beta diversity between outcomes was performed with permutational multivariate analysis of variance (PERMANOVA) with 10,000 iterations.

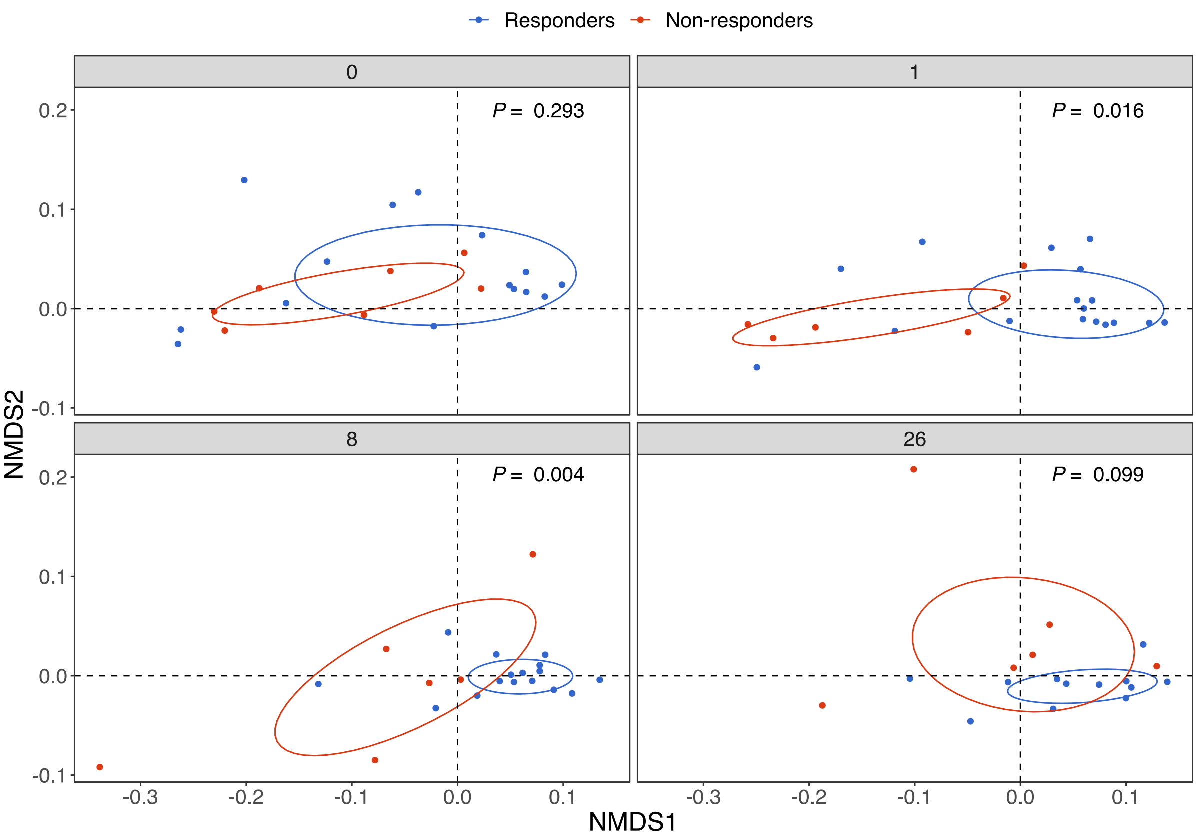

Figure S4. Beta diversity of functional potential estimated with PICRUSt2 based on Bray-Curtis distance and visualized with NMDS ordination. The analysis was performed with permutational multivariate analysis of variance (PERMANOVA) with 10,000 iterations.

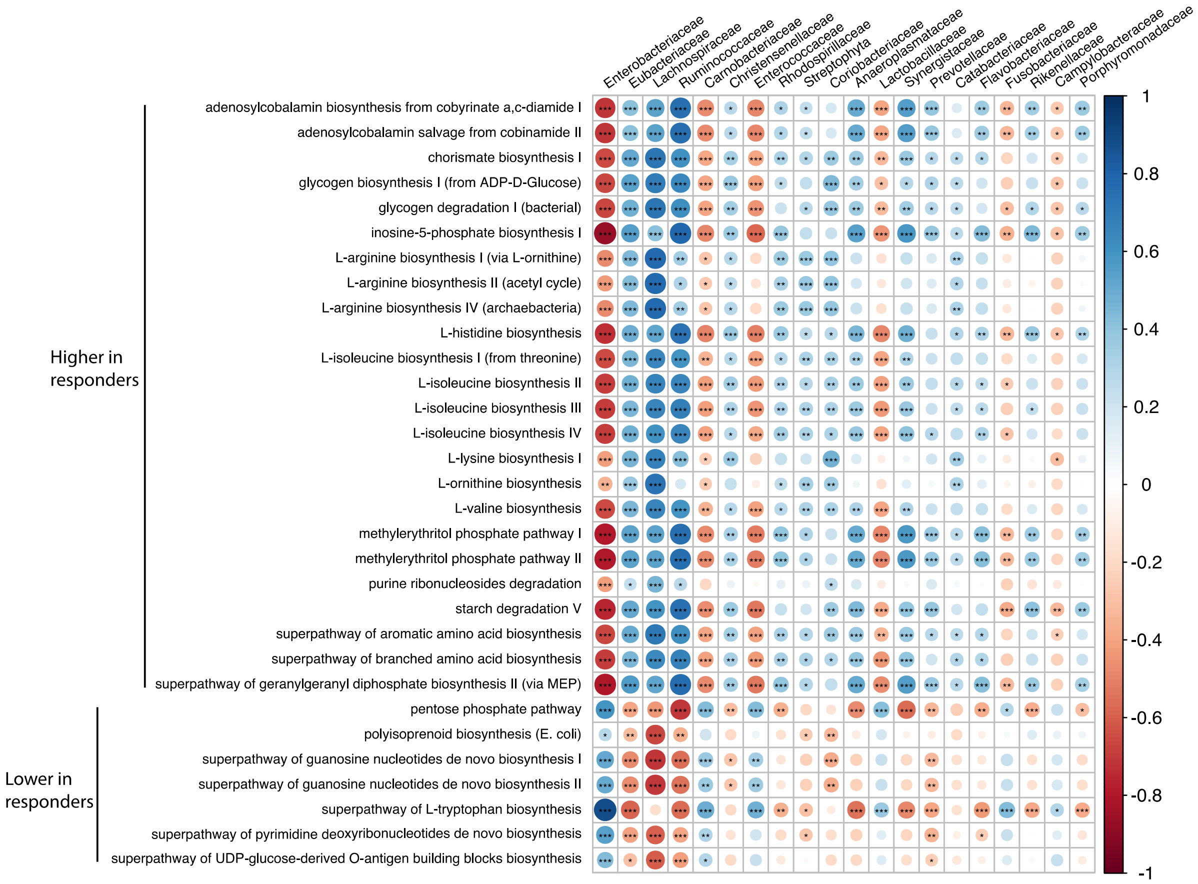

Figure S5. Correlations between the relative abundances of families and MetaCyc pathways. Blue color indicates positive Spearman's rank-order correlation coefficient and red color indicates negative correlations. The abundance of pathway labelled "Higher in responders" is more abundant in responders and the one labelled "Lower in responders" is less abundant in responders. The stars for each correlation refers to the significance level: *, $0.01 < P < 0.05$; **, $0.001 < P < 0.01$; ***, $P < 0.001$.

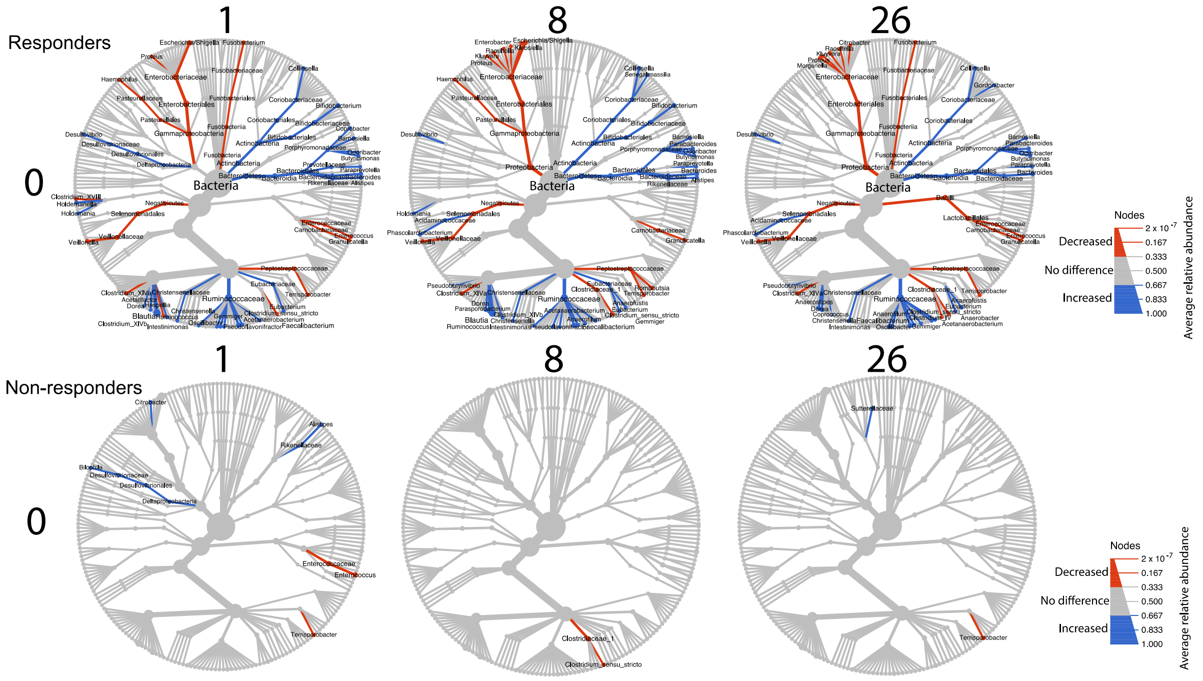

Figure S6. Tree plots of discriminant taxa as compared to pre-FMT. Comparisons were performed between post-FMT and pre-FMT samples within each outcome. The smallest nodes represent the genus level and the node in the center represents the kingdom bacteria. Node sizes are proportional to the mean relative abundance at the given phylogenetic level. Nodes with labels have significantly different relative abundances between time points, where the blue color indicates the taxa have increased over time, red color indicates decreased abundances, and grey color indicates no significant changes in abundances.

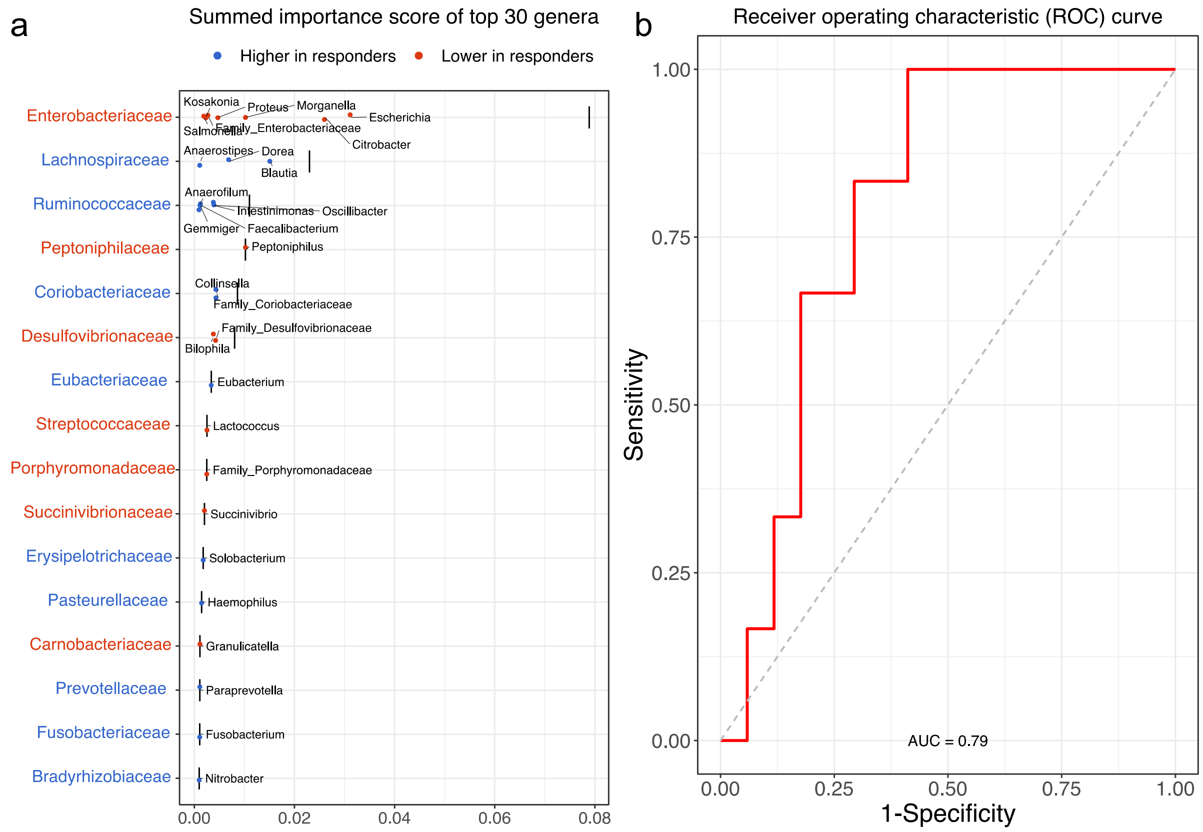

Figure S7. Importance and receiver operating characteristic (ROC) curve obtained by random forest modelling. (a) The top 30 most important genera are shown and their importance scores are summed (the black vertical line) based on the family they belonged to in descending order. Colors specify higher or lower abundances of corresponding families between outcomes. The variable 'importance' is obtained from the random forest model built based on the samples at week 1, *via* 10-fold cross validation with 100 iterations. (b) The ROC curve describing the ability of the model to differentiate outcomes when applied at different levels of sensitivity (true positive rate) and specificity (true negative rate). Area under the ROC curve (AUC) is used to measure the discriminatory power of the model (AUC 0.79).

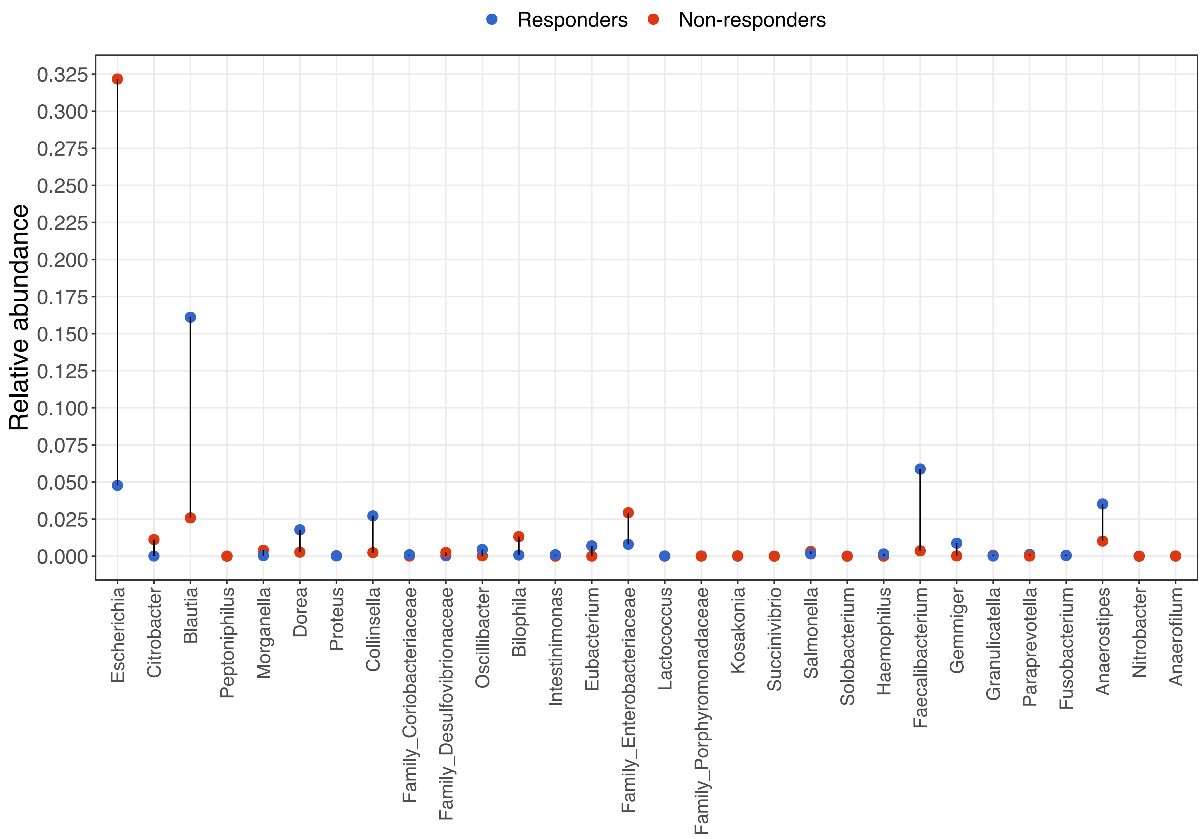

Figure S8. Relative abundances of the top 30 most important genera at differentiating outcomes identified by random forest modelling. The X axis lists the genera in a descending order from left to right according to importance. Colors refer to outcomes.

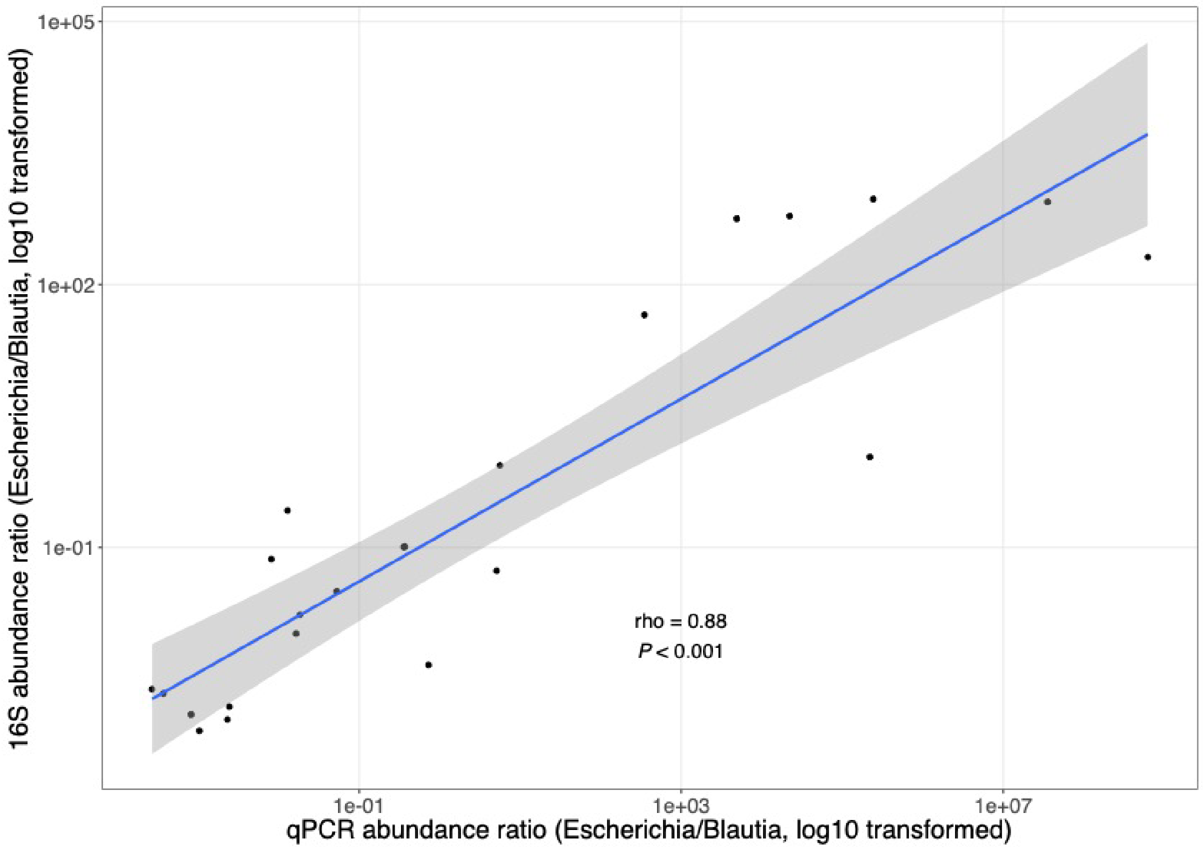

Figure S9. Correlation between the abundance ratios (*Escherichia* to *Blautia*) determined by qPCR and 16S. The X and Y axis is the abundance ratio determined by qPCR and 16S respectively, and log10 transformed for visualization. The strength of correlation is calculated using the non-parametric Spearman's rank-order correlation coefficient.

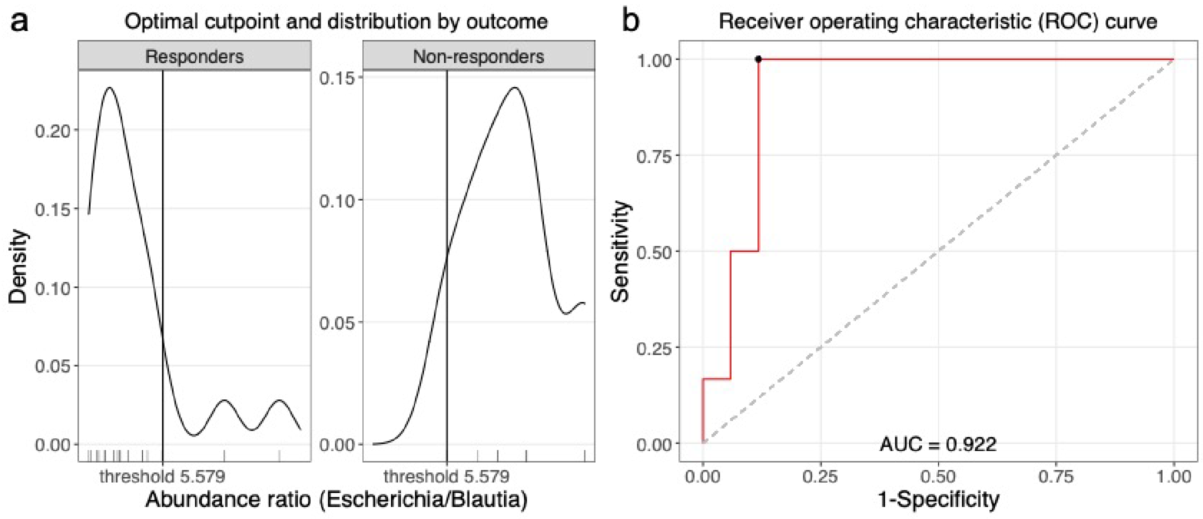

Figure S10. Prediction of outcomes based on the abundance ratio between *Escherichia* and *Blautia* at week 1. (a) The distribution of abundance ratios between genus *Escherichia* and *Blautia* for each outcome determined by qPCR. The optimal threshold of abundance ratios that best differentiated outcomes was obtained by the Youden's J statistic and calculated to be 5.6, where a larger value of abundance ratio than 5.6 is indicative of treatment failure. A prediction accuracy of 91.3% was obtained based on the chosen threshold. (b) The ROC curve describing the ability of the model to differentiate outcomes when applied at different levels of sensitivity (true positive rate) and specificity (true negative rate). Area under the ROC curve (AUC) is used to measure the discriminatory power of the model (AUC 0.922).

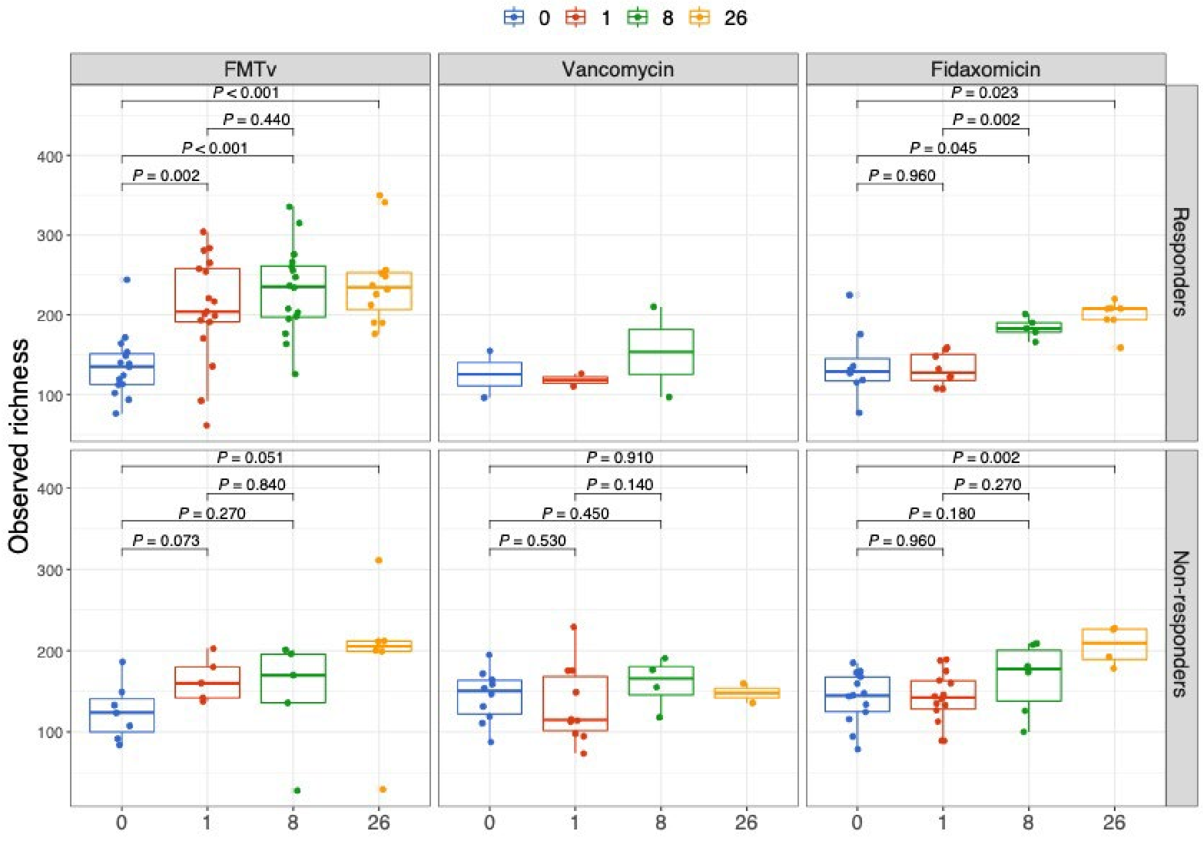

Figure S11. Observed richness for FMTv, vancomycin, and fidaxomicin treatments over time, stratified by outcomes. Boxplot is used to show the distribution of richness. The median richness is compared with the Wilcoxon rank sum test.

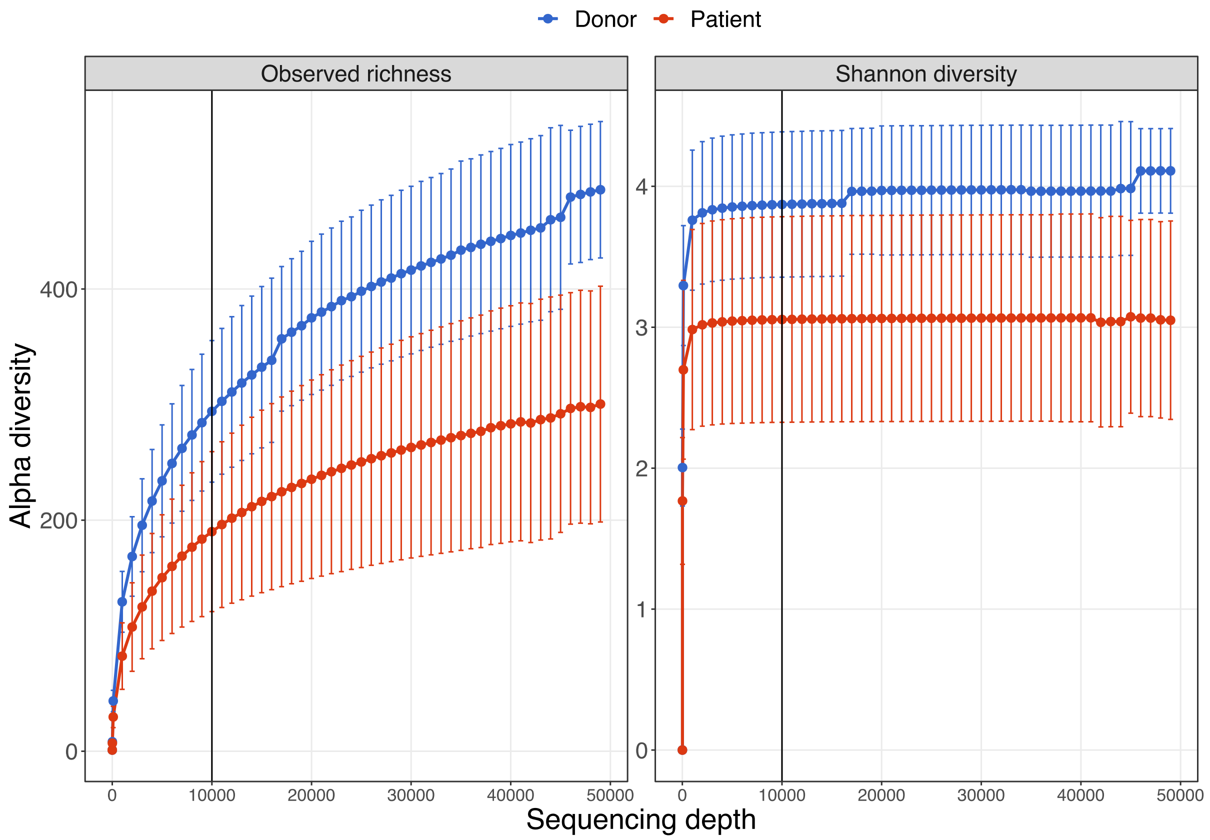

Figure S12. Rarefaction curve for observed richness and Shannon diversity for samples from the FMTv treatment. Donors (blue) are higher in alpha diversity than patients (red). Although the richness did not reach saturation before 50,000 reads, Shannon diversity was close to maximum after 2,000 reads. The black vertical lines mark the sequencing depth of 10,000 reads, which is the depth used for rarefaction before calculating alpha diversity. Dots represent the means of alpha diversity in corresponding sample types. Error bars show the standard deviation of alpha diversity obtained from 100 random rarefactions at the corresponding sequencing depth.

Table S1. Comparison of Bray-Curtis distance between time points or donors within each outcome for FMTv treatment.

Time	Outcome	P_{adj}
0-1	Responders	**0.002**
0-1	Non-responders	0.612
0-8	Responders	**0.002**
0-8	Non-responders	0.077
0-26	Responders	**0.002**
0-26	Non-responders	0.117
1-8	Responders	0.961
1-8	Non-responders	0.147
1-26	Responders	0.889
1-26	Non-responders	0.153
8-26	Responders	0.961
8-26	Non-responders	0.961
0-Donor	Responders	0.258
0-Donor	Non-responders	0.258
1-Donor	Responders	0.385
1-Donor	Non-responders	0.258
8-Donor	Responders	0.258
8-Donor	Non-responders	1.000
26-Donor	Responders	0.258
26-Donor	Non-responders	0.385

The comparison was performed between time points or between time and donor for each outcome. Permutational multivariate analysis of variance (PERMANOVA, 10,000 permutations) was used to test the comparison. P values adjusted with the Benjamini-Hochberg method are shown as P_{adj}. Significant P_{adj} values are in bold.

Table S2. Comparison of Bray-Curtis distances between time points within each outcome for vancomycin and fidaxomicin treatments.

Time	Outcome	Treatment	P_{adj}
0-1	Responders	Vancomycin	1.000
0-1	Non-responders	Vancomycin	0.441
0-8	Responders	Vancomycin	1.000
0-8	Non-responders	Vancomycin	0.541
0-26	Non-responders	Vancomycin	0.590
1-8	Responders	Vancomycin	1.000
1-8	Non-responders	Vancomycin	0.441
1-26	Non-responders	Vancomycin	0.674
8-26	Non-responders	Vancomycin	1.000
0-1	Responders	Fidaxomicin	1.000
0-1	Non-responders	Fidaxomicin	1.000
0-8	Responders	Fidaxomicin	0.749
0-8	Non-responders	Fidaxomicin	0.590
0-26	Responders	Fidaxomicin	0.541
0-26	Non-responders	Fidaxomicin	0.441
1-8	Responders	Fidaxomicin	1.000
1-8	Non-responders	Fidaxomicin	0.928
1-26	Responders	Fidaxomicin	0.830
1-26	Non-responders	Fidaxomicin	0.593
8-26	Responders	Fidaxomicin	1.000
8-26	Non-responders	Fidaxomicin	1.000

The comparison was performed between time points within each outcome. Permutational multivariate analysis of variance (PERMANOVA, 10,000 permutations) was used to test the comparison. *P* values adjusted with the Benjamini-Hochberg method are shown as P_{adj}.
